# MF59 Promoted the Combination of CpG ODN1826 and MUC1-MBP Vaccine-Induced Antitumor Activity Involved in the Enhancement of DC Maturation by Prolonging the Local Retention Time of Antigen and Down-Regulating of IL-6/STAT3

**DOI:** 10.3390/ijms231810887

**Published:** 2022-09-17

**Authors:** Jing Jie, Guomu Liu, Jingyue Feng, Desheng Huo, Yixuan Wu, Hongyan Yuan, Guixiang Tai, Weihua Ni

**Affiliations:** Department of Immunology, College of Basic Medical Science, Jilin University, Xinjiang Street 125, Changchun 130021, China

**Keywords:** MF59, CpG 1826, compound adjuvant, tumor vaccine, MUC1-MBP, dendritic cells

## Abstract

Our previous study found that CpG oligodeoxynucleotides 1826 (CpG 1826), combined with mucin 1 (MUC1)-maltose-binding protein (MBP) (M-M), had certain antitumor activity. However, this combination is less than ideal for tumor suppression (tumors vary in size and vary widely among individuals), with a drawback being that CpG 1826 is unstable. To solve these problems, here, we evaluate MF59/CpG 1826 as a compound adjuvant with M-M vaccine on immune response, tumor suppression and survival. The results showed that MF59 could promote the CpG 1826/M-M vaccine-induced tumor growth inhibition and a Th1-prone cellular immune response, as well as reduce the individual differences of tumor growth and prolonged prophylactic and therapeutic mouse survival. Further research showed that MF59 promotes the maturation of DCs stimulated by CpG1826/M-M, resulting in Th1 polarization. The possible mechanism is speculated to be that MF59 could significantly prolong the retention time of CpG 1826, or the combination of CpG 1826 and M-M, as well as downregulate IL-6/STAT3 involved in MF59 combined CpG 1826-induced dendritic cell maturation. This study clarifies the utility of MF59/CpG 1826 as a vaccine compound adjuvant, laying the theoretical basis for the development of a novel M-M vaccine.

## 1. Introduction

Mucin 1 (MUC1) is an attractive target in tumor immunotherapy. To date, four tumor vaccines targeting MUC1 have entered clinical trials: MUC1 peptide vaccine, antiMUC1 monoclonal antibody vaccine, MUC1-specific immune cell vaccine, and MUC1-expressing recombinant viral vector vaccine. The MUC1 protein vaccine induced T-cell proliferation and strong interferon (IFN) production [1]. Its obvious advantage lies in its high safety and low cost of production [2]. However, its immunogenicity is weak, so researchers havefocused on the development of adjuvants to enhance the immunogenicity of the vaccine [3]. Our group produced a MUC1-maltose binding protein (MBP) (M-M) recombinant fusion protein (constructed by inserting seven tandem repeats encoding the human MUC1. In a previous study, we found that the combination of the Toll-like receptors 9 (TLR9) agonist CpG oligodeoxynucleotides 1826 (CpG 1826) and M-M inhibited the growth of melanoma B16-MUC1 cells subcutaneously transplanted into mice and mediated MUC1-specific humoral and cellular immune responses [4]. However, CpG 1826 is a synthetic nonmethylated DNA sequence. Due to its instability and short half-life in the body, it has a short retention time in the body, so that the time of the antigen exposure to the immune cells is short, which reduces the antitumor effect. Thus, CpG 1826 in combination with the M-M vaccine is less than ideal for tumor suppression (tumors vary in size and vary widely among individuals). Moreover, its ability to induce MUC1-specific antibody production, Th1 response, CTL killing effect, BMDC on CpG 1826 uptake and DC maturation still need to be improved.

We tried to slow down the metabolic rate of CpG 1826 in vivo, promote CpG 1826 uptake by DCs, and more strongly induce the maturation of dendritic cells to promote CD4+ T cells to Th1 polarization. In this way, we could obtain a significant tumor suppressor effect, reduce individual differences, and prolong the survival of mice. Compound adjuvants have become a growing trend in the vaccine field to leverage their strengths and work together to promote one another’s effects [5]. MF59 is one of the first approved clinical adjuvants, composed mainly of squalene, Tween 80 and Span85, and clinical reports have shown extremely high safety and reliability since its marketing approval [6,7]. As an aqueous oil adjuvant, it can deliver antigen to drainage lymph nodes, enhance the surrounding antigen presenting cells to antigen uptake, and promote the dendritic cell differentiation of monocytes and granulocytes [8,9]. MF59 also enhances the Th1 reaction when combined with TLR4 and TLR9 adjuvants [10,11]. A recent antimelanoma experimental study showed that MF59 immunized mice with combined type C CpG ODN-YW002 produced very high levels of IgG2c and IFN-γ, inhibited MUC1-B16 melanoma growth, and prolonged the survival of tumor-bearing mice [12]. These results suggested that MF59 combined with CpG oligodeoxynucleotides might be developed as an effective adjuvant for tumor vaccines against melanoma.

In this study, to further optimize the vaccine effect, we screened adjuvants compatible with CpG 1826 from commonly used Al(OH)_3_, Bacillus Calmette-Guérin (BCG) and MF59, to enhance immunogenicity. Subsequently, we evaluated the immune-enhancing effect of MF59 and CpG 1826 as a compound adjuvant on M-M through tumor-bearing experiments and survival experiments. We also explored the possible mechanism of DC maturation and activation by MF59 in promoting CpG 1826 retention and downregulating the inhibition of the IL-6/STAT3 pathway to further promote the combination of M-M and CpG 1826. This study clarifies the mechanism of action of MF59/CpG 1826, reveals the role of both as compound adjuvants in the M-M vaccine, and demonstrates the application value of MF59/CpG 1826 as a vaccine compound adjuvant, laying the theoretical foundation for the development of a novel M-M vaccine.

## 2. Results

### 2.1. Adjuvants Used for Compatibility with CpG 1826 Were Screened: MF59 Promotes the Combination of M-M and CpG 1826-Induced MUC1-Specific Humoral and Cellular Immune Responses

**Figure 1:**
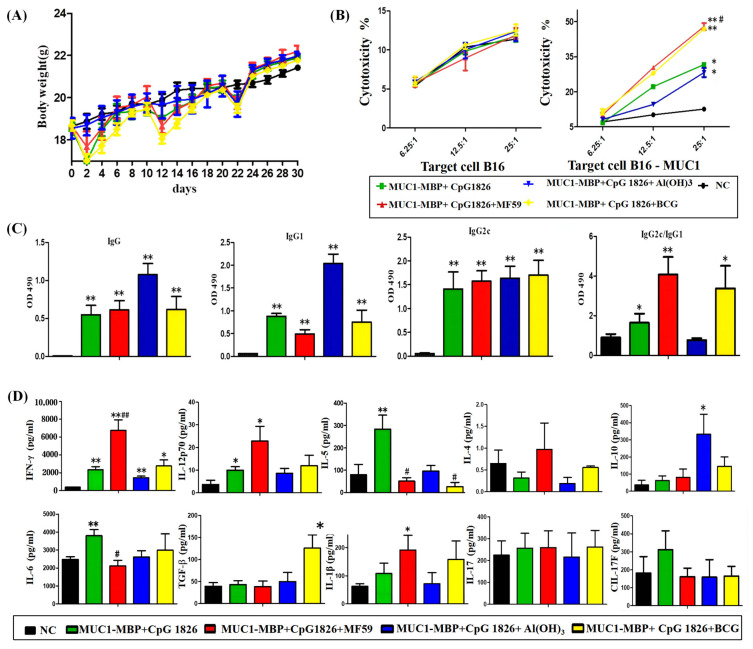
The effect of different adjuvant screenings in the combination of M-M/CpG 1826 on inducing MUC1-specific humoral and cellular immune responses. Mice were immunized with M-M/CpG 1826, M-M/CpG 1826/MF59, M-M/CpG 1826/Al(OH)_3_, M-M/CpG 1826/BCG or PBS once a week for a total of four times, in which the BCG-combination group was immunized twice (*n* = 10/group). The mice were killed four days after the last immunization. The sera were collected for the MUC1-specific antibody assay. The splenic mononuclear cells from each group were stimulated in vitro with a specific MUC1 peptide (20 µg/mL) for five days, and then a cell proliferation assay and cytokine assay were carried out. (**A**) Effects of different formulations of M-M combined with CpG 1826 on the body weight of mice. (**B**) Effects of different formulations of M-M combined with CpG 1826 on antibody subtypes in mice. (**C**) CTL killing activity on target B16 neo or B16-MUC1 cells. (**D**) The original image of the chip analysis and its expression in a bar graph of splenocyte cytokine secretion by the Quantibody^®^ array. Each mouse was replicated four times, and each group consisted of four mice. * *p* < 0.05, ** *p* < 0.01 vs. NC group. ^#^ *p* < 0.05, ^##^ *p* < 0.01 vs. the M-M/CpG 1826 group.

We coordinated CpG 1826 with MF59, aluminum adjuvant or BCG, respectively, and, together with M-M, immunized C57BL/6 mice. The body weight of each group of mice decreased slightly at 1–2 days after immunization (approximately 1–2 g) (Figure 1A). However, steady growth soon recovered, indicating that the mice were in good survival condition after immunization. The results showed that CpG 1826 combined successfully with MF59, Al(OH)_3_ and BCG on M-M.

To test whether vaccines with different compatibility adjuvants could induce MUC1-specific CTL killing in mice, isolated splenocytes were stimulated in vitro with MUC1 polypeptide for 5 days, used as effector cells, B16-MUC1 as target cells and examined for killing efficiency at different efficacy target ratios with B16-neo as a control. The results (Figure 1B) showed that both MF59/M-M/CpG 1826 and BCG/M-M/CpG 1826 immunization induced stronger MUC1-specific CTL activity compared with M-M/CpG 1826.

IgG, IgG1 and IgG2c are important subtypes in the humoral immune response of C57BL/6 mice. IgG1 represents an immune-prone response of the Th2 type, while IgG2c represents an immune-prone response of the Th1 type, and a higher IgG2c/IgG1 ratio indicates a more significant Th1 immune tendency. The results are shown in Figure 1C, showing that the MUC1-specific IgG antibody and the IgG1 antibody were significantly induced in each group, compared to the NC group. Meanwhile, a significant MUC1-specific IgG1 antibody was induced in each group, the most obvious effect seen in the Al(OH)_3_/CpG 1826/M-M group. The ratio of IgG2c/IgG1 in each group was higher than the ratio of IgG2c/IgG1 in the NC group, and the MF59/CpG 1826 group was significantly higher than the CpG 1826 group and the CpG 1826/BCG group, while the ratio in the CpG 1826 group was lower than the ratio in the control group. Each of the CpG 1826/M-M, and its combined adjuvant groups, appeared to be able to induce a MUC1-specific humoral immune response, while the Al(OH)_3_/CpG 1826/M-M group induced a Th2-prone response. The CpG 1826 group, MF59/CpG 1826 group, and CpG 1826/BCG group, produced a Th1-prone response, and the Th1 response was significantly higher in the MF59/CpG 1826 group than in the CpG 1826 group.

To further explore the effect of the compound adjuvant MF59/CpG 1826 and M-M on mouse Th subsets, splenocytes were isolated and restimulated in vitro with MUC1 polypeptide for 5 days, and the major cytokine levels of Th1, Th2, Th17 and Treg subsets were analyzed in the supernatant. The results are shown in Figure 1D. Compared with the M-M/CpG 1826 group, the IFN-γ and IL-12p70 levels of the compound adjuvant of MF59/CpG 1826 increased when combined with M-M, while IL-5 and IL-6 were downregulated. The Al(OH)_3_/CpG 1826/M-M produced significantly higher levels of IL-10 than those in each group. This suggested that the combination group induced a strong Th2 response. The TGF-β level produced was significantly higher in the BCG/CpG 1826/M-M group than in each of the other groups, suggesting that the BCG/CpG 1826/M-M group induced a Treg response. Neither IL-17 nor IL-17F changed significantly, suggesting that the MF59/CpG 1826/M-M group promoted a Th1 response, and had little effect on cytokines of the Th2, Th17 and Treg subsets.

### 2.2. MF59/CpG 1826 Combined with the M-M Enhances the Antitumor Effect by Inducing a Strong Th1 Response

#### 2.2.1. MF59 Promotes the Combination of M-M and CpG 1826-Inhibited B16-MUC1 Growth in Mice

**Figure 2:**
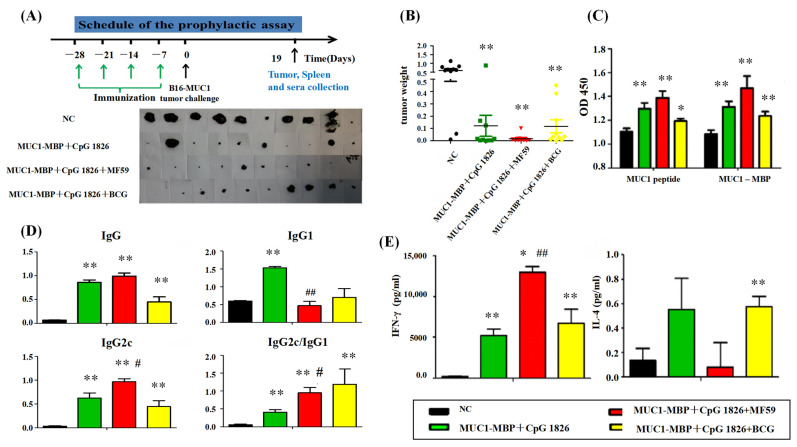
MF59 promotes the antimelanoma effect induced by M-M/CpG 1826. The mice were immunized with M-M/CpG 1826, M-M/CpG 1826/MF59, M-M/CpG 1826/BCG and PBS four times. One week after the final immunization, the mice were challenged with a subcutaneous injection (s.c.) with 5 × 10^5^/mouse B16-MUC1 cells to build a tumor-bearing model. (**A**–**C**) The immunization protocol in the prophylactic melanoma tumor model. The tumor and tumor weight in each vaccine formulation. Mice were sacrificed on Day 19, and tumors were harvested for weighing (*n* = 10). Effects of M-M combined with different adjuvants on lymphocyte proliferation (**D**) and cytokines (**E**) secreted by mouse spleen cells. * *p* < 0.05, ** *p* < 0.01 vs. NC group. ^#^ *p* < 0.05, ^##^ *p* <0.01 vs. the M-M/CpG 1826 group.

Analysis of tumor suppressor activity was grouped into NC group, M-M/CpG 1826, M-M/CpG 1826/MF59, M-M/CpG 1826/BCG. Immunization was performed according to the procedure shown in Figure 2A, and B16-MUC1 was administered 7 days after the last immunization to observe the tumor changes. On Day 19 after tumor inoculation, the tumor weight of each adjuvant group was significantly lower than the weight of the NC group (*p* < 0.01), especially the MF59/CpG 1826/M-M group, which had the best tumor suppression effect. More importantly, the tumor size and volume were homogeneous in the MF59/CpG 1826/M-M group (Figure 2A,B), suggesting MF59/CpG 1826/M-M could reduce the individual differences of tumor growth in the B16-MUC1 melanoma mouse model.

Subsequently, we analyzed the immunoactivity of tumor-bearing mice. The results showed that, the CpG1826/M-M group significantly stimulated the MUC1-specific proliferation of splenocytes, and the MF59 significantly promoted the MUC1-specific splenocyte proliferation induced by M-M/CpG1826 (Figure 2C), suggesting that splenocyte proliferation was antigen-dependent. When compared to the control, the secretion of IFN-γ was significantly higher in the MF59/CpG 1826/M-M group than in the CpG 1826/M-M group (*p* < 0.01) (Figure 2E), suggesting that the MF59/CpG 1826/M-M group induced the strongest Th1 response, consistent with the previous antitumor results, and suggested the role of the Th1 response in antitumor activity. Meanwhile, all the vaccine treated groups could induce MUC1-specific antibody (Figure 2D). The CpG1826/M-M group induced both Th1 and Th2, and the Th1 response was significantly lower in the CpG 1826/M-M group than the Th1 response of the MF59/CpG 1826/M-M group (*p* < 0.01), a trend consistent with the previous adjuvant screen.

#### 2.2.2. The Compound Adjuvant MF59/CpG 1826 Combined with the M-M Vaccine Significantly Prolonged the Survival of the Tumor-Bearing Mice

**Figure 3:**
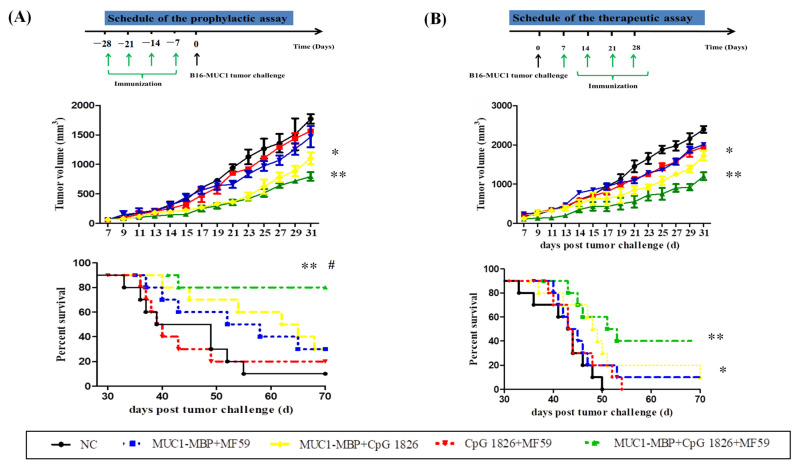
Antitumor effect of M-M combined with different adjuvants on survival in a prophylactic melanoma tumor model (**A**) and therapeutic model (**B**). (**A**) The immunization protocol in the prophylactic melanoma tumor Model. The tumor weight in each vaccine formulation (*n* = 10). The survival in each vaccine formulation. (**B**) Antitumor effect of M-M combined with different adjuvants in a therapeutic melanoma tumor model. The immunization protocol in the therapeutic melanoma tumor Model. The tumor weight in each vaccine formulation (*n* = 10). The survival in each vaccine formulation. * *p* < 0.05, ** *p* < 0.01 vs. NC group. ^#^ *p* < 0.05 vs. the M-M/CpG 1826 group.

To further compare the effects of MF59/CpG 1826/M-M on the survival of tumor-bearing mice, we performed a dynamic monitoring of survival in both prophylactic (Figure 3A) and therapeutic mouse models (Figure 3B). The results showed a similar trend in the effect of the combination of compound adjuvant on survival in the prophylactic and therapeutic treatment groups. The MF59/CpG 1826/M-M group had the slowest tumor growth rate. Next, tumor size in each group was analyzed at 31 days after tumor inoculation. A significant difference was found between the MF59/CpG 1826/M-M group and the NC group (*p* < 0.01). In the prophylactic group, the survival rate of the MF59/CpG 1826/M-M group remained at 80% at 70 days after the tumor injection, with no significant difference among the other groups (Figure 3A). In the therapeutic group, the survival rates of MF59/CpG 1826/M-M group and CpG 1826/M-M group at 50 days after tumor injection were 60% and 30%, respectively. At 70 days after tumor grafting, the survival rate of MF59/CpG 1826/M-M group remained at 40% at 70 days after the tumor injection. The above results suggested that the compound adjuvant MF59/CpG 1826, combined with the M-M, significantly prolonged the survival of the tumor-bearing mice.

### 2.3. MF59 Promotes CpG 1826/M-M-Induced Th1 Activation by Enhancing Dendritic Cell Maturation

#### 2.3.1. MF59/CpG 1826 Combined with the M-M Vaccine Significantly Promoted the Maturation of DCs in Lymph Nodes

**Figure 4:**
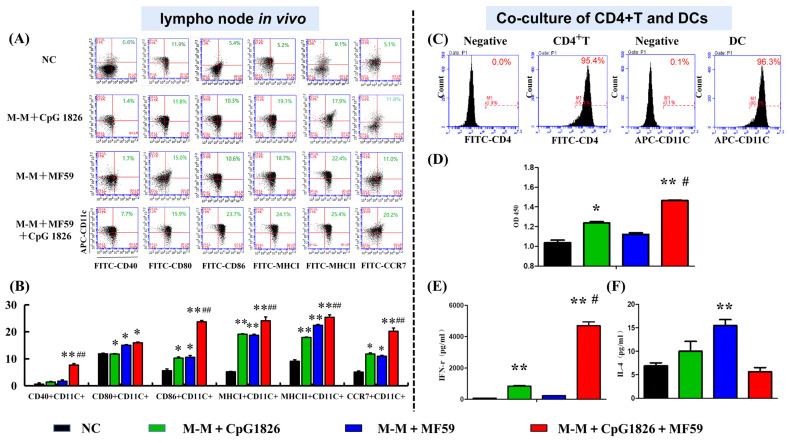
MF59 promotes CpG 1826- or M-M/CpG 1826-induced DC maturation, leading to Th1 activation. (**A**) The draining lymph node was isolated and s.c. injection of PBS, M-M/CpG 1826, M-M/MF59 and M-M/MF59/CpG 1826 in the flanks of C57BL/6 mice on day four with a two-week interval. The expression of maturation markers on LN DCs was analyzed by flow cytometry. (**B**) The percentage of DP cells is expressed as the mean ± standard deviation and is shown in a bar graph. (**C**–**E**) CD4+ T-cell activation is enhanced by coculture with BMDCs stimulated with the combination of MF59, M-M and CpG 1826. (**C**) The purity of CD4+ T cells from spleen samples from immunized mice and BMDCs from untreated mice was above 95%. (**D**) MF59/M-M/CpG 1826 synergistically increased the proliferation of cocultured CD4+ T cells and DCs. The production of IFN-γ (**E**) and IL-4 (**F**) in CD4+ T cells cocultured with DCs, as detected by ELISA. CD4 T cells were cocultured with DCs at a ratio of 50:1. All the experiments were repeated three times, and all the data are expressed as the mean ± SD (*n* = 5). Data were derived from three independent experiments. * *p* < 0.05, ** *p* < 0.01 vs. NC group. ^#^ *p* < 0.05, ^##^ *p* < 0.01 vs. the M-M/CpG 1826 group.

To further investigate the effect of MF59/CpG1826 on M-M-induced DC maturation in vivo, mouse lymph nodes were harvested after four immunizations of M-M/CpG 1826, M-M/MF59 and M-M/MF59/CpG 1826. Mouse lymph nodes were made into single-cell suspensions, and DC surface molecular expression was analyzed by flow cytometry. The results are shown in Figure 4A. In the MF59/CpG 1826/M-M group, except for a slight elevation in CD40+ (*p* < 0.05), the expression levels of other genes, such as CD86+, CCR7+, MHCΙ+ and MHCII+, were significantly increased (*p* < 0.01), more so than those in the other groups. This result suggested that MF59/CpG 1826 combined with M-M synergistically promoted the maturation of DCs in lymph nodes.

#### 2.3.2. MF59 Promotes CpG 1826/M-M-Induced th1 Polarization of CD4+ T Cells Cocultured with BMDCs

The purity of CD4+ T cells in BMDCs was identified by CD4+ T cells or CD11c cells using fluorescence activated cell sorting (FACS) (Figure 4C), both of which were above 95%.

To investigate whether the maturation of DCs promoted by MF59/CpG 1826 would affect the Th1 polarization of CD4+ T cells, the proliferation of cocultured DCs and CD4+ T cells was first detected using WST-1. The results showed that the compound adjuvant MF59/CpG 1826/M-M synergistically stimulated the proliferation of cocultured cells. MF59/CpG 1826/M-M promoted the Th1 polarization of CD4+ T cells cocultured with DCs (Figure 4D). Supernatants were harvested, and the levels of IFN-γ and IL-4 were measured by ELISA. Coculturing CD4+ T cells with BMDCs stimulated with MF59/CpG 1826/M-M significantly increased the production of IFN-γ, compared with the other groups (Figure 4E). At the same time, the level of IL-4 in MF59/CpG 1826/M-M group was lower than in the other groups (Figure 4F), suggesting the maturation and activation of DCs and the polarization of CD4+ T cells toward the Th1 phenotype.

### 2.4. The Effect of MF59 on CpG 1826 Uptake, Local Retention and Cell Localization

#### 2.4.1. MF59 Significantly Promoted DC Uptake of CpG 1826

**Figure 5:**
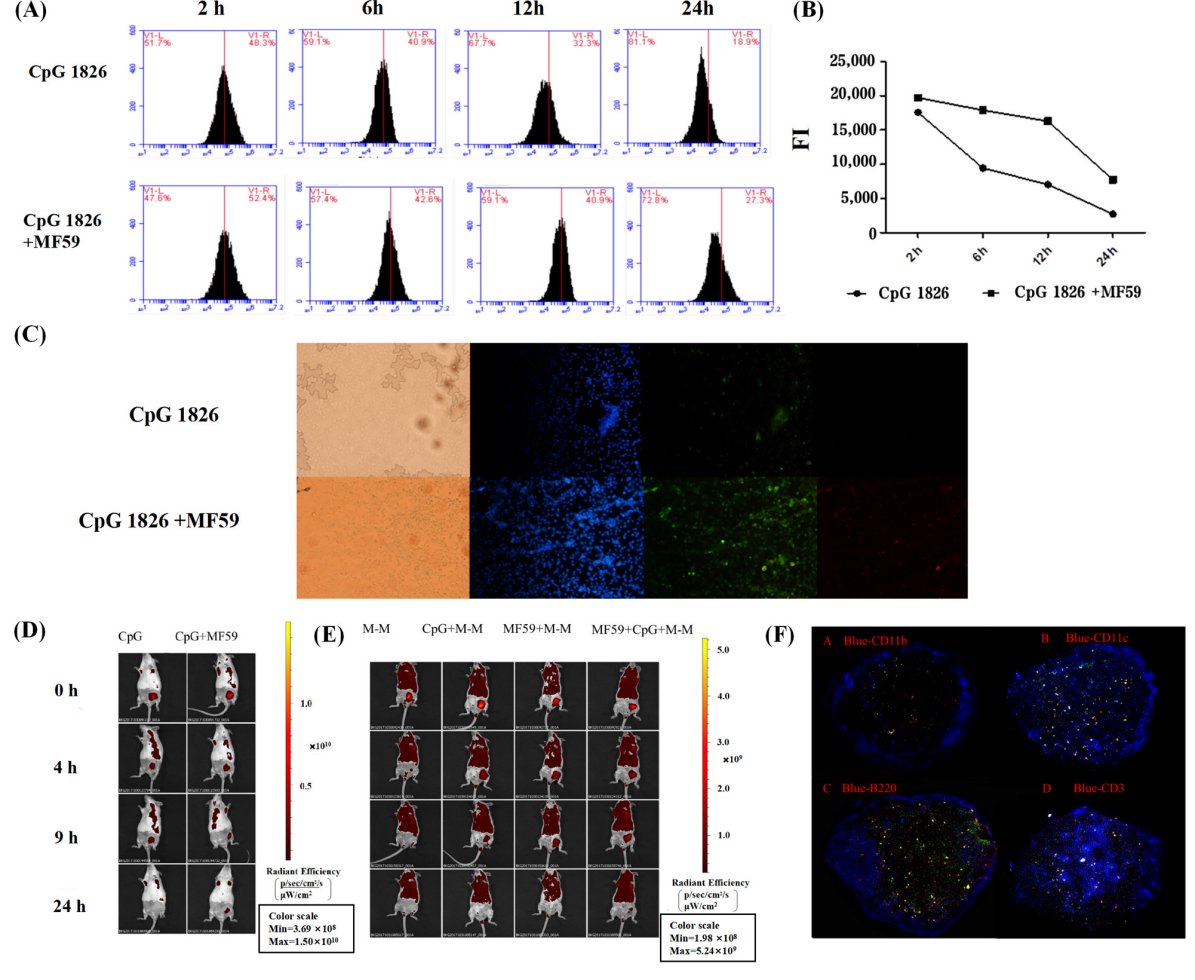
MF59/CpG 1826 promotes BMDC uptake, retention, and local retention in vivo and in vitro. (**A**–**C**) In vitro study. (**A**) Fluorescence intensity of CpG 1826-FITC-positive cells. Total fluorescence intensity (FI) = % of positive cells × mean fluorescence intensity. (**B**) MF59 promotes the uptake of CpG 1826 by BMDCs, as shown by fluorescence microscopy. Blue represents DAPI-stained nuclei, red represents Dil-labeled MF59, and green represents FITC-labeled CpG 1826. (**C**–**E**) In vivo study. (**C**) Effect of MF59 on CpG 1826 retention in mouse lymph nodes. Mice were immunized with FITC-labeled CpG or FITC-labeled CpG/MF59 and photographed under a small animal live imager at 0, 4, 9, and 24 h. (**D**) Effects of different adjuvants on the retention of M/M in mouse lymph nodes. Mice were immunized with FITC-labeled M-M, FITC-labeled M-M/CpG 1826, FITC-labeled M-M/MF59, or (**E**) FITC-labeled M-M/CpG1826/MF59 and photographed under a small animal live imager at 0, 4, 9, and 24 h. (**F**) Subcellular localization of CpG 1826 and MF59. Mice were immunized with FITC-labeled CpG 1826/Dil-labeled MF59. After 24 h of immunization, lymph nodes were taken to prepare frozen sections, and four slices were cut in the same site for immunofluorescence observation. Frozen sections of lymph nodes were stained with Violet-CD11b, Violet-CD11c, Violet-B220 and Violet-CD3. Yellow represents the colocalization of FITC-CpG and Dil-MF59. Data were derived from three independent experiments.

To investigate the effect of MF59/CpG 1826 on the uptake of BMDCs, the fluorescence intensity was analyzed by flow cytometry at 2 h, 6 h, 12 h and 24 h after stimulation with fluorescein isothiocyanate FITC-CpG 1826 or FITC-CpG 1826/Dil-MF59. The results showed that the fluorescence intensity of BMDCs in the MF59/CpG 1826 group was higher than the fluorescence intensity of BMDCs in the CpG 1826 group at each time point (Figure 5A,B) and peaked at 2 h, suggesting that MF59 combined with CpG 1826 significantly increased the uptake capacity of BMDCs.

Subsequently, the uptake of FITC-CpG 1826 and Dil-MF59 by BMDCs after 6 h was observed by fluorescence microscopy. The results showed that CpG 1826 entered the cells, and the uptake of CpG 1826 by DCs increased when CpG 1826 was combined with MF59, further confirming that MF59 combined with CpG 1826 significantly increased the uptake capacity of BMDCs (Figure 5C).

#### 2.4.2. MF59 Extended the Local Retention Time of CpG 1826/M-M in the Lymph Nodes

To investigate the effect of MF59 on the migration and retention of CpG 1826 in vivo, FITC-CpG 1826 was used. The mice were scanned by small animal in vivo imaging at 0 h, 4 h, 9 h, and 24 h after immunization. The results showed that after 9 h of immunization with CpG 1826 alone, the fluorescence at the lymph nodes was stronger but disappeared completely within 24 h. Partial fluorescence was still visible at 24 h when CpG 1826 was combined with MF59, suggesting that MF59 can prolong the retention of CpG 1826 in vivo (Figure 5D).

Subsequently, we investigated the effect of MF59/CpG 1826 on the migration and retention of M-M in mice. M-M labeled FITC was used. Mice were inoculated in subcutaneous inguinal combination with MF59, MF59, and MF59/CpG 1826 and scanned by small animal live imagers 0 h, 4 h, 9 h, and 24 h after immunization. The results showed that MF59/CpG 1826 significantly prolonged M-M retention in lymph nodes (Figure 5E).

#### 2.4.3. MF59/CpG 1826 Are Colocalized in Lymph Node DCs and T-Cell Regions

To further identify the cells where CpG 1826 and MF59 localized after immunization, we used FITC-labeled CpG 1826 and Dil-labeled MF59. The mice were inoculated subcutaneously in the groin in a volume of 100 μL and were euthanized 24 h after immunization. Lymph nodes were collected to prepare frozen sections. Four sections were continuously cut at the same position in the lymph nodes, and the following immune cells were stained: violet-CD3, violet-CD11b, violet-CD11c, and violet-B220. The results showed that most CpG 1826 overlapped with MF59 mainly in the CD11c+ cell region in the center of the lymph nodes (Figure 5F), which was also the main region of CD3+ T cells, suggesting that CpG 1826 combined with adjuvant MF59 might promote the uptake of antigen by DCs and presentation to T cells, which requires further research.

### 2.5. The MF59/CpG 1826 Compound Adjuvant Partly Promotes DC Maturation by Downregulating IL-6/STAT3

#### 2.5.1. MF59/CpG 1826 Significantly Downregulated the Expression of IL-6 at the mRNA Level

**Figure 6:**
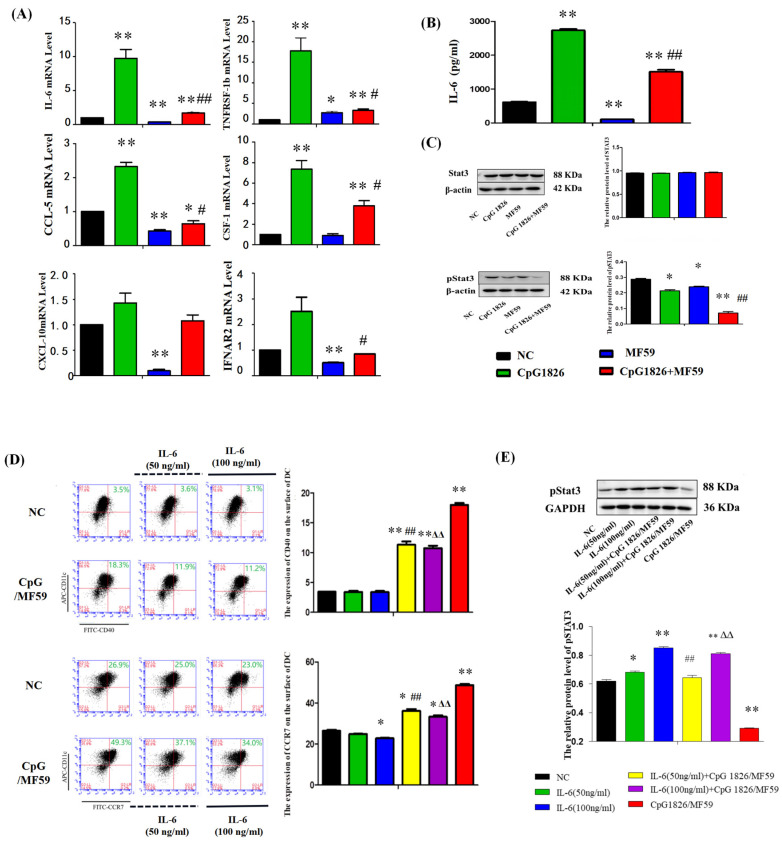
The MF59/CpG 1826 compound adjuvant partly promotes DC maturation by downregulating IL-6/STAT3. (**A**,**B**) qPCR and Western blot analysis of IL-6 mRNA and protein expression. BMDCs were stimulated with adjuvant CpG 1826 (5 μg/mL) and/or MF59 (1:400) for 48 h in vitro. The effects of CpG 1826 and MF59 on IL-6 mRNA and protein levels and other target gene mRNA levels (**A**,**B**), and the expression levels of STAT3 and pSTAT3 in BMDCs were analyzed by Western blotting (**C**). β-Actin was used as a control. (**D**,**E**) BMDCs were cultured in the absence or presence of IL-6 (50 ng/mL or 100 ng/mL) pretreatment for 3 days, and half of the liquid was replaced with IL-6 on the fifth day. On the sixth day, MF59/CpG1826 was added to the medium for another 48 h, and the concentrations of IL-4 and GM-CSF were maintained at 20 ng/mL. (**D**) The DC surface molecules CD40 and CCR7 were measured by flow cytometry. (**E**) The expression level of pSTAT3 in BMDCs treated with IL-6 was analyzed by Western blotting. GAPDH was used as a control. The relative level of pSTAT3 is expressed as the mean ± standard deviation and is shown in a bar graph. Data were derived from three independent experiments. * *p* < 0.05, ** *p* < 0.01 vs. NC group. ^#^ *p* < 0.05, ^##^ *p* < 0.01 vs. M-M/CpG 1826 group, ^∆∆^ *p* < 0.01 vs. MF59/CpG 1826 group.

MF59 and CpG 1826 were reported to be intramuscularly injected into mice and have a series of acting target genes, such as IL-6, CSF-1, CXCL10, TNFRSF-1β, IFNAR-2, IL-2, and IL-1β [13]. Our previous study also found that MF59/CpG 1826 could significantly downregulate IL-6 expression when compared with the CpG 1826 group (Figure 1D). Six identical target genes from MF59 and CpG 1826 were selected for qPCR detection: IL-6, CSF-1, TNFRSR-1b, IFNAR2, CCL-5, and CXCL-10. We isolated BMDCs and stimulated them with adjuvant for 48 h in vitro. The qPCR results showed that IL-6 secretion by CpG 1826 alone was extremely high but downregulated significantly when CpG 1826 was combined with MF59 (Figure 6A). IL-6 has been reported to inhibit DC maturation through the STAT3 signaling pathway [14,15]. Therefore, we used IL-6 as the entry point for further study.

#### 2.5.2. MF59/CpG 1826 Significantly Downregulated the Expression of IL-6 and pSTAT3

To further explore the DC maturation mechanism by MF59/CpG 1826, IL-6 secretion was analyzed by ELISA, and pSTAT3 was analyzed by Western blotting. The results showed that the expression of IL-6 (Figure 6B) and pSTAT3 (Figure 6C) was promoted by CpG 1826 alone but downregulated significantly by MF59/CpG 1826. These results suggested that the IL-6/STAT3 signaling pathway might be significantly downregulated by MF59/CpG 1826.

#### 2.5.3. IL-6 Significantly Inhibited the Promotion of MF59/CpG 1826 on the Maturation of DCs

To further validate the role of IL-6 in the maturation of DCs by CpG1826/MF59, IL-6 was used when DCs were stimulated with MF59/CpG 1826. The expression of DC surface markers was analyzed by flow cytometry. The expression of CD40 and CCR7 was significantly inhibited when IL-6 was pretreated. The expression rates of CD40 and CCR7 were lower in the IL-6 alone group than when combined with MF59/CpG 1826 in the same dose of IL-6, suggesting that MF59/CpG 1826 promoted the maturation of DCs and that maturation was inhibited by IL-6 (Figure 6D).

#### 2.5.4. IL-6 Reverses the Inhibition of pSTAT3 by MF59/CpG 1826

The expression of pSTAT3 was detected by Western blotting. The results showed that pSTAT3 expression could be inhibited by CpG1826/MF59. The inhibition status of pSTAT3 was reversed when CpG1826/MF59 cells were pretreated with IL-6. The expression level of pSTAT3 protein was promoted with increased IL-6 concentration. MF59/CpG 1826 is suggested to enhance the maturation of DCs through the inhibition of the IL-6/STAT3 signaling pathway (Figure 6E).

## 3. Discussion

The basic principles of tumor vaccine success include the delivery of large amounts of high-quality antigens to DCs, the induction of strong and sustained CD4+ T helper cells and cytotoxic T lymphocyte (CTL) responses through the activation of DCs, and the persistent infiltration of the TME [16]. Our previous study [4] found that CpG 1826 combined with the M-M vaccine could stimulate mouse spleen lymphocytes to produce strong Th1 activation, significantly prolonging the survival of mice, and had certain antitumor effects on melanoma, but there were still problems regarding unsatisfactory tumor suppression effects and large tumor differences in individual mice. CpG 1826 is a sequence of synthetic nonmethylated DNA sequences, and is characterized by instability. We needed to find a suitable adjuvant to slow down the metabolism of CpG 1826 in vivo and then increase the time of antigen exposure to immune cells. Aluminum and MF59 are the primary candidates for compound adjuvants and have been widely studied in influenza virus vaccine studies [17]. Our previous study showed that the antitumor vaccine M-M in combination with BCG induced the MUC1-specific Th1 immune response and cytotoxic T-cell killing activity and that it significantly inhibited the growth of MUC1-expressing B16 cells in mice [4]. Therefore, to further optimize the vaccine effect, Al(OH)_3_, BCG, and MF59 were first considered in this experiment. Through this study, we found that MF59/CpG 1826, as a compound adjuvant combined with M-M vaccine, induced a significant Th1 response and CTL killing, while CpG 1826/Al(OH)_3_ compound adjuvant combined with M-M induced a high level of Th2 immune response and a low level of Th1 response and CTL killing. CpG 1826/BCG combined with M-M induced both Th1 and Treg cell response and CTL killing. Taken together, the compound adjuvant MF59/CpG 1826 induced the best antitumor Th1 response and CTL killing. Therefore, we chose MF59 as a good candidate to coordinate with CpG 1826.

We further examined the effect of the MF59/CpG 1826 and M-M on the immune system and tumor suppression. The results showed that MF59/CpG 1826 tumor suppression was the most significant and homogeneous, which reflected the importance of selection of adjuvant for the immune response. MF59, CpG 1826 and M-M cooperated to produce a strong Th1-cell immune response, which was far better than the strength of the response generated by the combination of CpG 1826 and M-M. The survival results of the prophylactic and therapeutic studies showed that the MF59/CpG 1826 combined with M-M was better than CpG 1826 or MF59 alone combined with M-M, and we speculated that the water-wrapped oil nature of MF59 compensated for the disadvantage of fast CpG 1826 metabolism and prolonged the retention of the CpG 1826/M-M in mice. Therefore, we next analyzed the mechanism of the effect of the compound adjuvant vaccine on the maturation of dendritic cells.

DCs are key to initiating the adaptive immune response, and our previous study showed that CpG 1826 can activate DCs and induce an immune response in Th1 cells [4]. Similar studies reported that CpG 1826 enters DC cells via the lysosomal pathway and that uptake of CpG 1826 promotes DC maturation [18]. Therefore, we investigated the effect of MF59/CpG 1826 on DC maturation from in vivo lymph nodes. The results showed that MF59/CpG 1826/M-M promoted the expression of DC cell surface costimulatory molecules CD40, CD80, CCR7, and MHC molecules. Therefore, we speculated that the compound adjuvant MF59/CpG 1826/M-M might indirectly induce Th1 activation by synergistically promoting the maturation and activation of dendritic cells. We further confirmed that MF59/CpG 1826/M-M promoted CD4+ T-cell Th1 polarization by inducing dendritic cell maturation.

Furthermore, we explored the specific mechanisms by which the combination of MF59/CpG 1826 and M-M affects dendritic cell maturation and activation. On the one hand, in vitro studies found that the fluorescence intensity of the antigen within BMDCs in the MF59/CpG 1826 group was significantly higher than the fluorescence intensity in the CpG 1826 group, suggesting that MF59 promoted the CpG 1826 uptake capacity of BMDCs. Small animal live imaging experiments revealed that MF59 significantly prolonged CpG 1826 and M-M in lymph node local retention time, indicating that MF59 plays a cell library role, slowly releasing into the CpG 1826 microenvironment, to overcome the disadvantages of short half-life, and increase the time of antigen exposure to immune cells. MF59/CpG 1826 promoted lymph node DC antigen intake, and better antigen delivery to T cells. This process was confirmed by the immunofluorescence results: after immunization, most CpG 1826 and MF59 colocalized in the region of DCs and T cells at 24 h after immunization.

On the other hand, the literature has reported that CpG 1826 alone induced high expression of pSTAT3, and the combined application of pSTAT3 inhibitors and CpG 1826 significantly inhibited tumor growth [19,20]. STAT3 activation is important for maintaining immune tolerance [20]. Interleukin 6 (IL-6) has been reported to inhibit the differentiation maturation of DCs via the viability of STAT3 [21,22,23,24]. IL-6 is the same target gene of MF59 and CpG 1826, which inhibits DC maturation through the STAT3 signaling pathway [25]. Our results showed that BMDCs significantly increased IL-6 secretion and STAT3 activation after CpG 1826 stimulation, which was significantly downregulated when CpG 1826 was combined with MF59. Further, the inhibition status of pSTAT3 and the upregulation of CCR7 and CD40 by CpG1826/MF59 was reversed when cells were pretreated with IL-6. CCR7 and CD40 were key cytokines in DC maturation [26]. CCR7 affected the migration ability of DCs and the CTL killing effect [27]. These results suggested that MF59/CpG 1826 promoted DC maturation and activation and might be associated with downregulating of IL-6/STAT3.

## 4. Materials and Methods

### 4.1. Materials

Total sulfur modification CpG 1826 (TCCATGACGTTCCTGACGTT) was commissioned by Sangon Biotech (Shanghai, China). Al(OH)_3_ was purchased from Changchun Institute of Biological Products Company (Changchun, China). BCG was purchased from Founding Company (Shijiazhuang, China). Span-85, Tween-80 and squalene were purchased from Shanghai Aladdin Biochemical Technology Company (Shanghai, China), DingGuochangSheng Biotechnology Company (Beijing, China), and Tixi Aicheng Industrial Development Company (Shanghai, China), respectively. IMDM medium and RPMI-1640 medium were purchased from Gibco (Carlsbad, CA, USA). Fetal BBS and mouse lymphocyte isolate solution were purchased from Tianjin Haoyang Biological Manufacture Company (Tianjin, China). The BCA protein concentration detection kit was purchased from Beijing Beyotime Biotechnology Company (Beijing, China). The PE-conjugated goat anti-mouse IgG antibody, the mouse IFN-γ, IL-4, IL-6 and IL-12p70 ELISA kits, as well as anti-mouse MHC, CCR7, CD3, and CD4 antibodies, were purchased from eBioscience (San Diego, CA, USA).

### 4.2. Cell Lines

B16-MUC1 cells were constructed and used to establish a tumor model, as described previously [4]. Briefly, murine B16 melanoma cells were transfected with the pcDNA3-MUC1 VNTR plasmid encoding the human MUC1 VNTR peptide and selected in complete medium containing G418 (600 mg/L) (Sigma-Aldrich, St. Louis, MO, USA) for B16-MUC1 cell clones, and the stable monoclonal transfected cells were verified by a flow cytometer (BD BioSciences, San Jose, CA, USA).

### 4.3. Preparation of MF59 and Dil-MF59

The MF59 emulsion was prepared by mixing Tween 80, Span 85, squalene and 10 NM sodium citrate at volume ratios of 0.5% (2.5 mL), 0.5% (2.5 mL), 4.3% (21.5 mL) and 94.7% (473.5 mL). The samples were centrifuged at 6000 RPM for 5 min without stratification, that is, to reach the standard of emulsification, and then the membranes were filtered to remove bacteria. The effective particle size was 7.7 nm, the degree of homogeneity was 0.229: a degree of homogeneity <0.7 was good. The particle size was tested again 6 months after preparation. The particle size was stable, and MF59 could be used for subsequent experimental studies.

Dil was mixed with squalene at a ratio of 20 μL/mL, and Span 85, Tween 80, squalene, and sodium citrate were added at the ratio described above. They were subsequently mixed and emulsified for 15 min.

### 4.4. Animals

C57BL/6 mice (6–8 weeks old, weighing 18–20 g) were purchased from HFK Bioscience Co. (Beijing, China). Mice were allowed free access to a laboratory chow diet and water. Animal handling procedures were conducted in accordance with the National Institute of Health Guide for the Care and Use of Laboratory Animals and conducted with the permission of the Ethics Committee of Jilin University (No. 2015-34).

### 4.5. Experimental Design in Mice

#### 4.5.1. Adjuvant Screening Experiments

The mice were randomly divided into five groups (10 mice/group): NC group (receiving PBS), M-M (50 μg)/CpG1826 (50 μg), M-M/CpG1826 (50 μg)/MF59, M-M/CpG1826 (50 μg)/Al(OH)_3_ (1.0 mg), M-M (50 μg)/CpG1826 (50 μg)/BCG (1.0 mg). The mice were immunized once a week in the groin and the back of the neck (totally 200 μL) for a total of four injections, in which the BCG-combination group did not receive BCG from the third immunization (totally two injections). The mice were killed four days after the last immunization.

Sera were isolated from the immunized mice, and the MUC1-specific antibodies were determined by ELISA. The spleen was taken to calculate the spleen index = spleen weight/body weight (calculated from the last weight). Splenic mononuclear cells were isolated using Ficoll from mice and cultured in Iscovs’s modified Dubecco’s medium (IMDM) containing 100 U/mL interleukin-2 with or without 20 µg/mL MUC1 synthetic peptide at a density of 1 × 10^6^ cells/well at 37 °C in 5% CO_2_ for five days in 96-well plates. Cell supernatants were collected for cytokine analysis by a Quantibody^®^ Array (Liaoning Baihao Biotechnology Co., Ltd., Tieling, China); the cells were used for the MUC1-Specific CTL Cytotoxicity Assay.

#### 4.5.2. Tumor Protection in a Prophylactic Model

a. Tumor growth inhibition. The mice were randomly divided into four groups (10 mice/group): NC group (PBS), M-M (50 μg)/CpG 1826 (50 μg), M-M (50 μg)/CpG 1826 (50 μg)/MF59, M-M (50 μg)/CpG 1826 (50 μg)/BCG (1.0 mg). The immunizing method was as described above. One week after the final immunization, the mice were challenged with a s.c. injection with 5 × 10^5^/mouse B16-MUC1 cells to build a tumor-bearing model. The inoculation site was the right sub-axillary.

Tumor size was measured by calipers, and the tumor volume (mm^3^) was calculated using the following formula: (a × b^2^)/2, where a is larger and b is smaller than the two measurements. Mice were sacrificed at Day 19 after tumor implantation. Sera were isolated from the immunized mice, and the MUC1-specific antibodies were determined by ELISA. Splenic mononuclear cells were isolated and treated as described above. Cell supernatants were collected for cytokine analysis by ELISA; the cells were used for lymphocyte proliferation assays.

b. In the survival test, a total of 50 mice were divided into 5 groups (*n* = 10). The immunization method and dosage were the same as above, while the groups were PBS, MF59/CpG 1826 (50 μg)/M-M (50 μg), CpG 1826 (50 μg)/M-M (50 μg), MF59/M-M (50 μg), and MF59/CpG 1826 (50 μg). One week after the final immunization, B16-MUC1 melanoma cells (5 × 10^5^/mouse) were injected into the back of the neck. The weight was recorded with a Vernier caliper on alternate days, and survival was monitored as described previously [4].

#### 4.5.3. The Therapeutic Model

The groups of mice were as follows (*n* = 10): NC group, MF59/CpG 1826 (50 μg)/ M-M (50 μg), CpG 1826 (50 μg)/M-M (50 μg), MF59/M-M (50 μg), MF59/CpG 1826 (50 μg) groups. The immunization method and dosage were the same as above. Mouse B16-MUC1 melanoma cells (5 × 10^5^/mouse) were injected into the back of the neck. At 7 days after tumor injection, the mice were immunized with different adjuvant vaccines containing M-M 4 times for 4 weeks. The weight was recorded with a Vernier caliper, and survival was monitored on alternate days.

### 4.6. MUC1-Specific CTL Cytotoxicity Assay

MUC1-specific spleen cells prepared above were used as the effective cells. B16-MUC1 or B16-neo cells were used as the target cells, and 1 × 10^4^ cells/well were added to the U-96-well plate. The effective cells and target cells were cocultured for 4 h at 37 °C and 5% CO_2_, and the ratio of E/T = 25:1, 12.5:1 or 6.25:1. The supernatant (50 μL/well) was transferred to another new 96-well plate. An LDH kit (Promega Corporation, Madison, WI, USA) was used to analyze the cytotoxicity. The 490 nm absorbance was detected. The percent cytotoxicity was calculated as follows: Cytotoxicity (%) = (effectors and target mixture − effectors spontaneous − target spontaneous)/(target maximum − target spontaneous) × 100%.

### 4.7. ELISA for MUC1-Specific Immunoglobulin Subclasses

ELISA analysis was performed as described previously [4]. Briefly, 96-well plates were coated overnight at 4 °C with 10 µg/well. After blocking with PBS containing 2% bovine serum albumin, the sera prepared above (in Section 4.5.1 and 4.5.2) were diluted 1:500 and later incubated at 37 °C for 1.5 h. After washing three times, HRP-labeled goat anti-mouse IgG, IgG1 and IgG2c (Sigma Chemical Co., St. Louis, MO, USA) were used as the detection antibody and incubated for 1 h at 37 °C. Then, the substrate *o*-phenylenediamine dihydrochloride (OPD, Amresco, Solon, OH, USA) was added after washing and later incubated for 10 min. Then, 0.2 mM H_2_SO_4_ was added to terminate the reaction. The absorbance was detected using an automated microtiter plate reader (BioTek Instruments, Inc., Winooski, VT, USA).

### 4.8. Cytokine Assay

#### 4.8.1. MUC1-Specific Th-Secreting Cytokines by Quantibody^®^ Array

A Quantibody^®^ array was used to analyze the changes in cytokines (IFN-γ, IL-12p70, IL-4, IL-5, IL-6, IL-10, TGF-β, IL-17A, IL-17F and IL-1β). The performance was conducted as described previously [4], according to the instructions. The secretion was analyzed by the cytokine–antibody–biotin complex and was visualized by the addition of the streptavidin-conjugated Cy3 equivalent dye using a laser scanner.

#### 4.8.2. ELISA

An ELISA kit (eBioscience, San Diego, CA, USA) was used to analyze the markers (IFN-γ, IL-4, IL-6 and IL-12p70) according to the manufacturer’s instructions. Each well was measured at 450 nm absorbance.

### 4.9. Isolation of BMDCs

BMDCs were isolated and cultured, as previously described in [4]. Briefly, cells (2 × 10^6)^ derived from the femurs of C57BL/6 mice were seeded into a six-well plate with RPMI 1640 supplemented with 10% fetal calf serum (FCS), 20 ng/mL IL-4 (PeproTech, London, UK), and 20 ng/mL Granulocyte-macrophage colony stimulating factor (GM-CSF) (PeproTech). The cells were harvested on Day 6 and analyzed by flow cytometry before they were used.

### 4.10. Analysis of DC Maturation

The mice were randomly divided into four groups of 5 animals. The mice were subcutaneously immunized with M-M (50 µg)/CpG 1826 (50 µg), M-M (50 µg)/MF59, or M-M (50 µg)/MF59/CpG 1826 (50 µg) four times with a two-week interval. Five days after the final immunization, the mice were sacrificed. The draining lymph nodes were made into a single-cell suspension with a 200-mesh filter. The maturation of cells was analyzed by a flow cytometer.

### 4.11. Coculture of BMDCs and CD4+ T cells

Spleen samples were obtained from each group (Section 4.10), and the mononuclear cells were separated using Ficoll. CD4+ T cells were isolated from this single-cell suspension using the CD4+ T-Cell Isolation Kit II, an LS column, and a MidiMACS™ separator (Miltenyi Biotec, Bergisch Gladbach, Germany). Non-CD4+ T cells were indirectly magnetically labeled by using a cocktail of biotin-conjugated antibodies (10 µL/10^7^ total cells) and anti-biotin microbeads (20 µL/10^7^ total cells). The harvested CD4+ T cells were incubated with a FITC-labeled antiCD4 antibody to determine the purity by flow cytometry before they were used.

CD4+ T cells (1 × 10^7^/mL) and BMDCs (2 × 10^5^ cells/mL) were cocultured at a ratio of 50:1, added to a 96-well plate, and then treated with medium containing M-M/CpG 1826 (5 µg/mL), M-M/MF59 (1:400) or M-M/CpG 1826/MF59 (5 µg/mL). The ability of T cells to proliferate and secrete IFN-γ and IL-4 was determined by WST-1 and ELISA, respectively.

### 4.12. MF59 and CpG 1826 Uptake, Local Retention and Cell Localization

#### 4.12.1. Fluorescence Microscopy Analyzed MF59/CpG 1826 on DC Uptake

Mouse BMDCs were seeded at a density of 1 × 10^6^ cells/mL in 12-well plates and placed in a CO_2_ incubator overnight. On the next day, FITC-CpG 1826 (5 μg/mL). alone or combined with Dil-MF59 (1:400), was added and stimulated for 24 h. Each well was fixed with 4% paraformaldehyde for 10 min and incubated with 100 μL diamidino-2-phenylindole (DAPI) at 4 °C for 5 min. The uptake of CpG 1826 and MF59 by BMDCs was observed by fluorescence microscopy (Olympus, Tokyo, Japan).

#### 4.12.2. Flow Cytometry Detecting MF59/CpG 1826 on DC Uptake

The operation was performed as above, and the fluorescence intensity was analyzed by flow cytometry at 2 h, 6 h, 12 h and 24 h after stimulation with FITC-CpG 1826 (5 μg/mL), alone or combined with MF59 (1:400). Total fluorescence intensity (FI) = % of positive cells × mean fluorescence intensity, and FI was used to compare the difference in the total amounts of FITC-CpG 1826 entering the cells at different times.

#### 4.12.3. Local Retention and Migration Analysis of MF59, CpG 1826 and M-M in the Lymph Nodes

To investigate the effect of MF59 on the migration and retention of CpG 1826 or M-M in vivo, the mice were scanned by small animal in vivo imaging (Maestro CRI Company, Woburn, MA, USA) (excitation peak 490 nm, emission peak 525 nm) at 0 h, 4 h, 9 h, and 24 h after immunization with FITC-CpG 1826 (5 μg/mL), alone or combined with MF59 (1:400).

The experiments were performed as described above, but immunization with FITC-M-M alone, or in combination with CpG 1826 and/or MF59, was performed.

#### 4.12.4. Colocalization of MF59/CpG 1826 in Lymph Node DCs and T-Cell Regions

The mice were inoculated subcutaneously in the groin in a volume of 100 μL and were euthanized 24 h after immunization with FITC-CpG 1826/Dil-MF59. Lymph nodes were collected to prepare frozen sections. Four sections were continuously cut at the same position in the lymph nodes, and the following immune cells were stained: violet-CD3, violet-CD11b, violet-CD11c, and violet-B220. The localization of CpG and MF59 was observed by fluorescence microscopy.

### 4.13. Analysis of the Effect of the Composite Adjuvant MF59/CpG 1826 on IL-6/STAT3 Signaling in DCs

BMDCs were prepared by isolation as described above and cultured in vitro stimulated with CpG (5 μg/mL), MF59 (1:400), CpG (5 μg/mL) plus MF59 (1:400) for 48 h, and mRNA expression of IL-6 was measured by qRT-PCR. Culture supernatant was analyzed by ELISA to analyze the effects of CpG 1826, MF59 and MF59/CpG 1826 on the secretion of IL-6 protein by DCs and Western blot analysis measured pSTAT3 expression.

To further investigate the role of IL-6 in MF59/CpG 1826 in promoting DC maturation, we pretreated BMDCs with exogenous IL-6. BMDCs were cultured in the absence or presence of IL-6 (50 ng/mL or 100 ng/mL) pretreatment for 3 days, and half of the liquid was replaced with IL-6 on the fifth day. On the sixth day, CpG 1826 (5 μg/mL), MF59 (1:400),CpG 1826 (5 μg/mL) plus MF59 (1:400) was added to the medium for another 48 h, and the concentrations of IL-4 and GM-CSF were maintained at 20 ng/mL. Cells were harvested, and the DC surface molecules, CD40, and CCR7, were measured by flow cytometry. STAT3 and pSTAT3 expression in BMDCs were measured by Western blotting.

### 4.14. FACS

Lymph node mononuclear cells, BMDCs or CD4+ T cells, prepared as described above, were incubated for 30 min at 4 °C with FITC-CD40, FITC-CD80, FITC-CD86, FITC-MHCI, FITC-MHCII, PE-CCR7, and FITC-CD4. Isotype control antibodies were used to estimate nonspecific binding of the target primary antibodies. Cells were analyzed using a flow cytometer.

### 4.15. WST-1 for Proliferation

WST-1 reagent was added to each well at a final concentration of 10% (*v*/*v*), and the plate was cultured in the dark at 37 °C for 1 h. Next, the absorbance was measured using a microplate reader at a wavelength of 450 nm. The results were shown as the relative cell viability. The relative cell viability was calculated as A450 (MUC1-stimulated group)/A450 (control group).

### 4.16. QRT-PCR

Total RNA was extracted from the cells using an RNAsimple Total RNA Kit (centrifugal column type) (Tiangen Biochemical Technology Limited Company, Beijing, China), according to the manufacturer’s instructions. CDNA was transcribed using a TransScript First-Strand cDNA Synthesis SuperMix Kit (TransGen, Beijing, China), in accordance with the manufacturer’s instructions. PCR was performed on the Opticon 2 Real-Time PCR Detection System (Bio-Rad, Hercules, CA, USA) by using the Power SYBR Green PCR Master Mix (Roche Diagnostics, Basel, Switzerland). Samples were run in duplicate and normalized to β-actin using the 2^–ΔΔCt^ method. The primer sequences and reaction parameters are shown in Table 1. The expression levels of the mRNAs were then reported as fold changes versus control.

### 4.17. Western Blot

BMDCs, prepared as described above (Section 4.13), were lysed with radio immunoprecipitation assay (RIPA) lysis buffer, and the protein concentration was obtained using the bicinchoninic acid protein detection system (Bio-Rad, Hercules, CA, USA). Proteins were separated using sodium dodecyl sulfate-polyacrylamide gel electrophoresis (SDS-PAGE) and subjected to Western blotting. AntiSTAT3 and antiSTAT3 were used, and β-actin was used as an internal control. Signals were detected using an electrochemiluminescence (ECL) reagent (Amersham, Piscataway, NJ, USA).

### 4.18. Statistical Analysis

Data was subjected to one-way analysis of variance (ANOVA) (SPSS 18.0 software. Chicago, IL, USA). When the main experimental results were statistically significant, a two-sided T test was performed. Survival was assessed by the Kaplan-Meier method and was assessed using a logarithmic test. *p*-values < 0.05 were considered significant.

## 5. Conclusions

Elucidation of the mechanisms involved in the combination of adjuvants for tumor vaccines is important for the development and application of these vaccines. In this study, we found that the MF59/CpG 1826 vaccine, combined with the M-M vaccine, participates in antitumor activity by inducing a robust Th1 response. The MF59/CpG 1826 compound adjuvant combined with M-M more significantly induced a Th1 response specific to the treatment and CTL killing in MUC1 versus the adjuvant CpG 1826/M-M. Tumor-bearing mice showed that MF59 significantly promoted CpG 1826/M-M induced Th1 response, significantly promoted the tumor suppressor effect, significantly reduced the individual differences of tumor growth, and significantly prolonged the survival of tumor-bearing mice. The mechanism might be that MF59 prolonged the local retention time of CpG 1826/M-M in lymph nodes, and MF59/CpG 1826 also inhibited the IL-6/STAT3 pathway, leading to the promotion of maturation of dendritic cells and, then, the promotion of Th1 differentiation. This study demonstrated the utility of MF59/CpG 1826 as a vaccine compound adjuvant, laying the theoretical basis for the development of a novel M-M vaccine. Further study will focus on the potential benefits of different delivery pathways, which remain to be fully elucidated.

## Figures and Tables

**Table 1:** Primer sequences and reaction parameters used for qRT-PCR analysis.

Gene	Primer
CXCL-10	Forward:5′-GCCGTCATTTTCTGCCTCA-3′
	Reverse: 5′-CGTCCTTGCGAGAGGGATC-3′
CSF-1	Forward:5′-CCAATGCTAACGCCACCGAGAG-3′
	Reverse:5′-CCTTGTTCTGCTCCTCATAGTCCTTG-3′
IFNAR2	Forward:5′-GTGCCGCTGCTCAGACTACATC-3′
	Reverse: 5′-CTCGTGCTTCTTCCTAACGCTGTC-3′
IL-6	Forward:5′-AGACTTCCATCCAGTTGCCTTCTTG-3′
	Reverse: 5′-CATGTGTAATTAAGCCTCCGACTTGTG-3′
CCL-5	Forward:5′-CCGCACCTGCCTCACCATATG-3′
	Reverse: 5′-CACACTTGGCGGTTCCTTCGAG-3′
TNFRSF-1b	Forward:5′-CTGCTGATGTTAGGACTGGTGAACTG-3′
	Reverse: 5′-TCAACAGGTGCTGCTGCTCAAG-3′

## Data Availability

Not applicable.

## Abbreviations

MUC1-MBP	Recombinant mucin1-maltose-binding protein
M-M	MUC1-MBP
DC	Dendritic cell
MBP	Maltose-binding protein
CpG	CpG Oligodeoxynucleotides
B16-MUC1	Human MUC1-overexpressed mouse melanoma B16 cells
CTL	Cytotoxic T lymphocyte
BCG	Bacillus Calmette-Guérin
TME	Tumor microenvironment
MUC1	Mucin-1
